# Diffusion and catalyst efficiency in hierarchical zeolite catalysts

**DOI:** 10.1093/nsr/nwaa184

**Published:** 2020-08-21

**Authors:** Peng Peng, Xiong-Hou Gao, Zi-Feng Yan, Svetlana Mintova

**Affiliations:** State Key Laboratory of Heavy Oil Processing, China University of Petroleum, Qingdao 266580, China; Laboratory of Catalysis and Spectrochemistry (LCS), Normandy University, National Graduate School of Engineering of Caen (ENSICAEN), University of Caen (UNICAEN), French National Center for Scientific Research (CNRS), Caen 14000, France; Petrochemical Research Institute, China National Petroleum Company, Beijing 100195, China; State Key Laboratory of Heavy Oil Processing, China University of Petroleum, Qingdao 266580, China; State Key Laboratory of Heavy Oil Processing, China University of Petroleum, Qingdao 266580, China; Laboratory of Catalysis and Spectrochemistry (LCS), Normandy University, National Graduate School of Engineering of Caen (ENSICAEN), University of Caen (UNICAEN), French National Center for Scientific Research (CNRS), Caen 14000, France

**Keywords:** diffusion, effectiveness factor, pore connectivity, hierarchical zeolite, industrial catalyst, advanced characterization

## Abstract

The preparation of hierarchical zeolites with reduced diffusion limitation and enhanced catalyst efficiency has become a vital focus in the field of zeolites and porous materials chemistry within the past decades. This review will focus on the diffusion and catalyst efficiency of hierarchical zeolites and industrial catalysts. The benefits of diffusion and catalyst efficiency at two levels of hierarchies (zeolitic component level and industrial catalyst level) from a chemical reaction engineering point of view will be analysed. At zeolitic component level, three types of mesopores based on the strategies applied toward enhancing the catalyst effectiveness factor are presented: (i) ‘functional mesopores’ (raising effective diffusivity); (ii) ‘auxiliary mesopores’ (decreasing diffusion length); and (iii) ‘integrated mesopores’ (a combination thereof). At industrial catalyst level, location and interconnectivity among the constitutive components are revealed. The hierarchical pore interconnectivity in multi-component zeolite based industrial catalysts is exemplified by fluid catalytic cracking and bi-functional hydroisomerization catalysts. The rational design of industrial zeolite catalysts at both hierarchical zeolitic component and catalyst body levels can be fully comprehended using the advanced *in situ* and/or operando spectroscopic, microscopic and diffraction techniques.

## INTRODUCTION

Zeolites are crystalline porous materials consisting of tetrahedrally coordinated elements (Si, Al, P, Ti, etc.) connected by oxygen bridge bonds [1]. Owing to the unique crystalline structure, variable chemical composition, high thermal stability and surface acidity, zeolites are widely used in petroleum refining, petrochemical, fine chemical and environmental catalysis [2,3]. However, for reactions where bulky molecules with diameters greater than the micropores of zeolites are involved, the mass transport of reactants from the outer surface to the acidic active sites experiences strong diffusion resistance, leading to reduced catalyst efficiency (*vide infra*). Furthermore, even if the reactants are small enough to enter the internal volume of zeolites, diffusion resistance may also extend the contact time with the acidic active sites within the crystals [4,5], which can lead to side reactions and deactivation [6–8]. Therefore, enhancing the efficiency and improving the catalytic cracking conversion of zeolites is one of the most urgent issues for both academia and industry [7,9].

A theoretical analysis from a chemical reaction engineering aspect can bring some solutions for the above-mentioned issue. Based on the law of mass action, the rate of reaction is proportional to the concentration of reactants. However, due to the diffusion resistance, the concentration near the surface of the catalyst usually deviates from the concentration at the acidic active sites. Therefore, two descriptors are proposed for quantitative measurement of catalyst efficiency in chemical reaction engineering [10–15].

The first descriptor is Thiele modulus (*ϕ*), which is a dimensionless number defined according to Equation (1):

(1) \begin{equation*} \varphi = L\sqrt {\frac{{kc_{{\rm{As}}}^{n - 1}}}{{{D_{{\rm{eff}}}}}}} , \end{equation*}

where *L*, *D*_eff_, *n*, *c*_As_ and *k* are diffusion length (m), effective diffusivity (m^2^ s^−1^), order of the reaction (dimensionless), concentration at the acidic active sites (mol m^−3^) and intrinsic reaction rate constant based on catalyst volume (unit of *k* depends on *n*), respectively. Here, *L* and *D*_eff_ indicate the contribution of diffusion rate, while *k*, *c*_As_ and *n* describe the reaction rate at the active sites. In this sense, *ϕ* is a quantitative index used to evaluate the relative magnitude between reaction rate at the active sites and the diffusion rate within the catalyst.

For a pseudo-first order reaction (e.g. catalytic cracking), the expression of *ϕ* can be simplified according to Equation (2):

(2) \begin{equation*} \varphi = L\sqrt {\frac{k}{{{D_{{\rm{eff}}}}}}} . \end{equation*}

The second descriptor, the catalyst effectiveness factor (*η*), is expressed according to Equation (3) below:

(3) \begin{equation*} \eta = \frac{{{r_{\rm{o}}}}}{{{r_{\rm{i}}}}}, \end{equation*}

where *r*_o_ (mol m^−3^ s^−1^) and *r*_i_ (mol m^−3^ s^−1^) represent observed and intrinsic reaction rates, respectively. Because the mutual exchange rate between influent and effluent reaction species on the surface of catalysts is not infinite [16,17], *r*_o_ is always lower than *r*_i_, leading to *η* of <1.0, but the closer *r*_o_ is to *r*_i_, the highest the *η* is. Therefore, introducing mesopores into parent zeolites (microporous structures) to prepare hierarchical porous materials with improved accessibility of active sites is a feasible way to enhance *η*.

**Figure 1.**
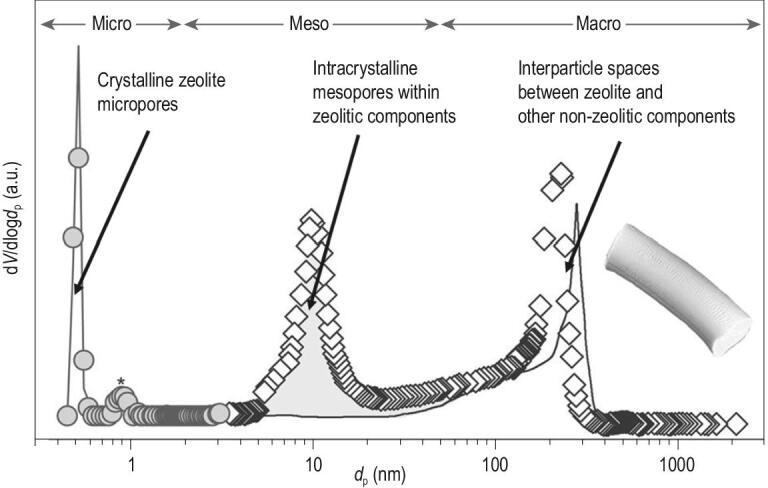
Pore size distribution of mesoporous (solid circle and hollow diamond) and conventional ZSM-5 zeolite (solid line) contained industrial catalyst bodies. Re-drawn and adapted with permission from [19].

In real industrial catalytic processes, the zeolitic component alone (even with hierarchical structure) cannot satisfy all the requirements [18]. As shown in Fig. 1 [19], the multi-component zeolite based catalyst intrinsically has a micro-/macroporous hierarchical structure consisting of both zeolitic microporosity and non-zeolitic macroporosity. If the zeolitic component is a hierarchical zeolite, additional mesopores will be integrated and form a micro-/meso-/macroporous tri-modal hierarchical catalyst body. Obviously, there are two levels of hierarchies for a zeolite multi-component catalyst body, i.e. (i) the hierarchy at zeolitic level, and (ii) the hierarchy at industrial catalyst level. In the following section of this review, we will reveal the synthesis, characterization and diffusion aspects of industrially used multi-component hierarchical zeolite catalysts at both levels.

## RATIONAL DESIGN OF HIERARCHY AT ZEOLITIC LEVEL: FUNCTIONAL VS. AUXILIARY MESOPORES

The preparation of hierarchical zeolites has become a focus in the field of zeolite and porous material science within the past decades. In light of the improvement on catalyst efficiency for diffusion-controlled catalytic reaction, the classification of the mesopores is of vital importance.

The relationship between *η* and *ϕ* is expressed as follows:

(4) \begin{equation*} \eta = \frac{{{\rm{tanh}}\varphi }}{\varphi }. \end{equation*}

For catalysts bearing strong diffusion resistance (*η* < 0.25), *ϕ* is reciprocal of *η*, i.e.:

(5) \begin{equation*} \eta = \frac{1}{\varphi }. \end{equation*}

Combining Equation (2) and Equation (5), Thiele modulus can be calculated using the following equation:

(6) \begin{equation*} \frac{1}{\eta } = \varphi = L\sqrt {\frac{k}{{{D_{{\rm{eff}}}}}}} . \end{equation*}

**Figure 2.**
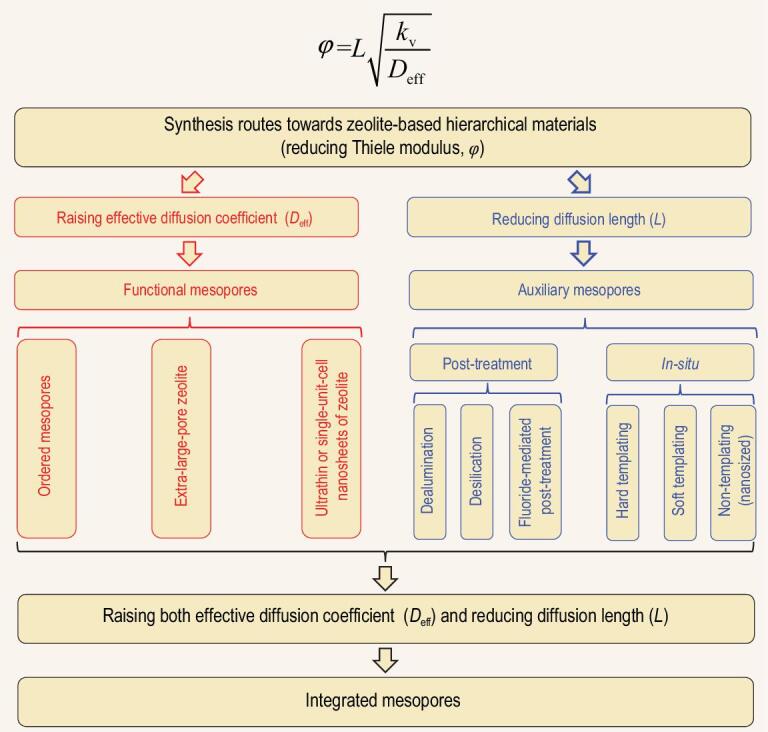
Overview of the synthesis routes toward zeolite-based hierarchical materials.

If the number of acidic active sites and other parameters are not changing, *k* can be regarded as a constant. In order to decrease *ϕ* for improving *η*, the two strategies proposed are: (i) enhancing effective *D*_eff_ or (ii) decreasing *L* [20]. Three types of hierarchical zeolite structures with the following mesopores (i) ‘functional mesopores’, (ii) ‘auxiliary mesopores’ and (iii) ‘integrated mesopores’ will be described (Fig. 2).

### Functional mesopores

**Figure 3.**
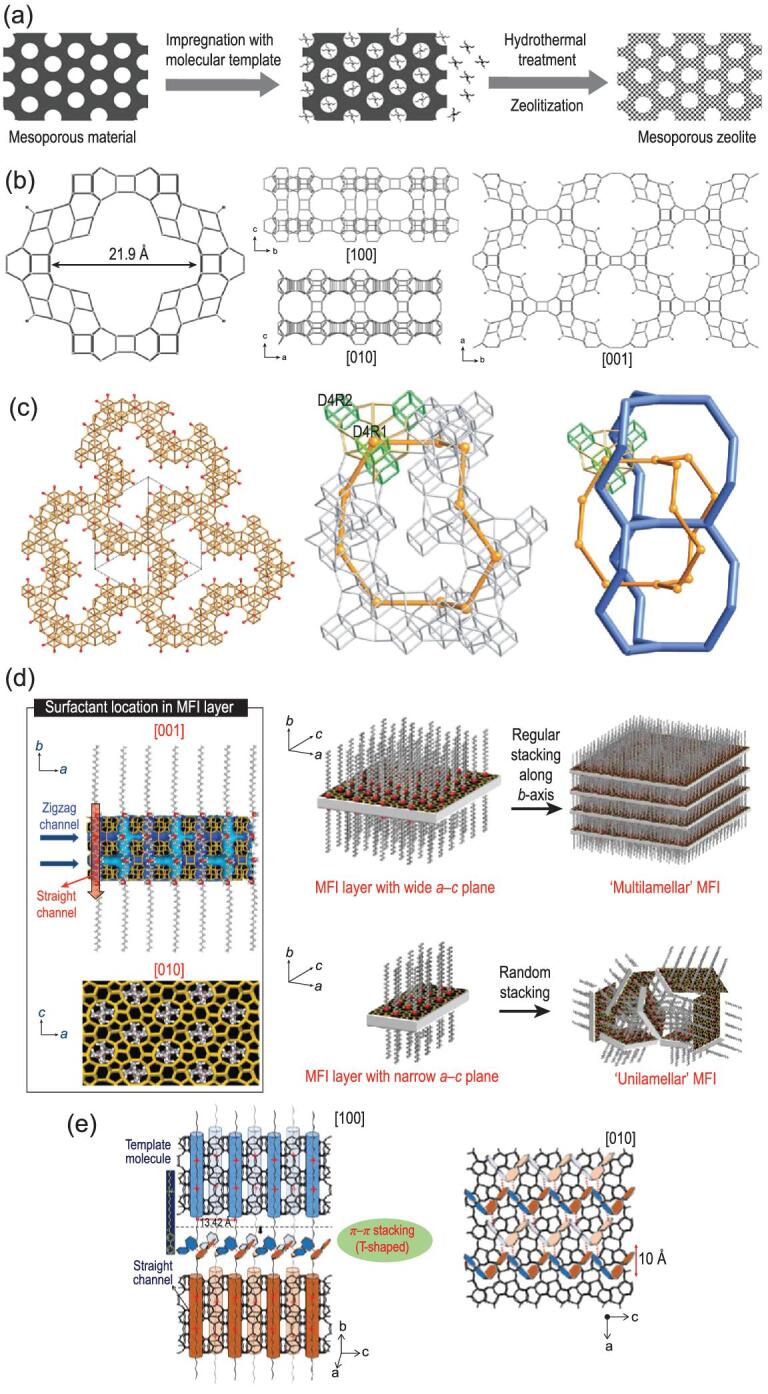
Representative functional mesopores. (a) Zeolitized ordered mesoporous material. Adapted with permission from [27]. (b) Framework of ITQ-43. Adapted with permission from [33]. (c) Framework of ITQ-37. Adapted with permission from [34]. (d) Crystallization of hierarchical single-unit-cell nanosheets of zeolite MFI. Adapted with permission from [37]. (e) Single quaternary ammoniums in the template molecules are located in the straight channel and serve as a template to direct the formation of hierarchical single-crystalline mesostructured zeolite nanosheets. Adapted with permission from [44].

If the thickness of mesopore wall is at the scale of several unit cells, the diffusion constrain within microporous structures is negligible. For these type of hierarchical zeolitic materials, the zeolitic layers are so thin that they can be directly regarded as mesopore walls that include acid active sites on their surface. In this regard, the mesopores are called functional mesopores. Owing to the wide mesopore walls, the diffusion type changes from configurational diffusion to surface or Knudsen diffusion [21], and the *D*_eff_ increases by several orders of magnitude, leading to a great enhancement of catalyst effectiveness. The mesopores in: (i) ordered mesoporous materials; (ii) extra-large-pore zeolites; and (iii) ultra-thin or single-unit-cell zeolite nanosheets are typical examples of functional mesopores (Fig. 3).

Ordered mesoporous materials like M41S [22] and SBA-*x* [23] contain functional mesopores. Due to their arranged mesoporous arrays, defined pore size distributions and tuneable surface properties, these materials have been used in reactions where bulky molecules are involved [24–26]. However, there are distinct differences between the ordered mesoporous materials and zeolites. The silica/alumina species are lacking long-range order in the mesopore walls, making them similar to ordinary amorphous silica/alumina. Due to the amorphous nature of the mesopore walls, these materials have low hydrothermal stability, which is a drawback for catalytic applications. Aiming at modifying the amorphous nature of the mesopore walls and improving their hydrothermal stability, researchers have proposed several methods to alter ordered mesoporous materials. Zeolitization of the mesopore walls is a typical example (Fig. 3a) [27]. During zeolitization of the mesopore walls, a template-like cetyl trimethyl ammonium bromide (CTAB) was added to a traditional zeolite synthesis precursor mixture containing tetra propyl ammonium bromide (TPABr) as structure directing agent [28]. Under hydrothermal treatment, some of the amorphous silica-alumina species in the mesopores can be transformed into primary and secondary building units of zeolite, leading to enhanced hydrothermal stability of the final material.

The components responsible for cracking and maximizing low-carbon olefins are due to the presence of FAU type zeolite (pore diameter and framework density (FD) of 0.74 nm and 13.3 T-atom/nm^3^, respectively) and MFI type zeolite (pore diameter and FD of 0.54 nm and 18.4 T-atom/nm^3^, respectively). If the pore diameter of the zeolites can reach the mesoporous scale (greater than 2 nm), the gap between micro- and mesopores can be sealed. In such cases, the mesoporous structure will exhibit better mass transfer performances and maintain acidity and hydrothermal stability similar to zeolites. The connections of extra-large-pores via four-membered rings and even three-membered rings result in structures with higher distortion tension. Si-O-Si or Si-O-Al bonds cannot withstand large distortion tension, which decreases the overall stability of the zeolite framework. The synthesis of extra-large-pore zeolites was successfully performed using germanium (Ge) with an atomic diameter higher than silicon, so Ge atoms can resist the distortion tensions [29,30]. Following this line of thought, Corma *et al.* reported the synthesis of a series of new type Ge-contained zeolites [31–34]. The ITQ-43 consists of a 28-member ring structure with FD of 11.4 T-atom/nm^3^. The largest pore aperture of ITQ-43 reached 2.19 nm, making it the first micro-/mesoporous hierarchical zeolite with long-range ordering at atomic scale (Fig. 3b) [33]. Although ITQ-37 has a slightly lower pore aperture (1.93 nm) than ITQ-43, its FD was further reduced to 10.3 T-atom/nm^3^, making it one of the zeolite frameworks with the lowest FD to date (Fig. 3c) [34]. The important role of pre-designed organic structure directing agent (OSDA) for synthesizing extra-large-pore zeolites has to be elaborated carefully. Recently, Zhang *et al.* reported an extra-large-pore germanosilicate zeolite SYSU-3 with novel SYT-type framework using a new type of OSDA via modifying alkaloids extracted from traditional Chinese herbal medicine [35].

Applications of ordered mesoporous materials in reactions involving bulky molecules are limited due to their low hydrothermal stability originating from the amorphous mesopore walls [24,36]. In order to cope with these problems, more targeted synthesis toward preparation of single-unit-cell [37–39], hierarchical pentasil zeolite nanosheets [40,41] and delamination of zeolites [42] were developed. The single-unit-cell ZSM-5 nanosheets, which were firstly reported by Ryoo's group are typical cases [37–39]. They designed an amphiphilic surfactant with two quaternary ammonium nitrogen atoms (C_22_H_45_-N^+^(CH_3_)_2_-C_6_H_12_-N^+^(CH_3_)_2_-C_6_H_13_, C_22-6-6_). In the C_22-6-6_ molecule, the hydrophilic N^+^(CH_3_)_2_-C_6_H_12_-N^+^(CH_3_)_2_-C_6_H_13_ head is served as OSDA for forming the MFI framework, while the hydrophobic C_22_H_45_- tail is used for separating the above single-unit-cell crystals. Due to the limited length of hydrophilic head (∼2 nm), the MFI framework structure can only be confined within a single-unit-cell scale, forming ultra-thin layered structures (Fig. 3d). The direct interconnection between hydrophilic head and hydrophobic tail via covalent C-C bond guarantees alternately arranged MFI layer and template chain layer of a size of 2.8 nm. Compared with the traditional ZSM-5, the single-unit-cell ZSM-5 nanosheets showed higher conversion in the cracking reaction of high density polyethylene. In methanol-to-gasoline reaction, the sample also showed better coking resistance than the traditional ZSM-5. Further, the Ryoo group investigated different surfactant with three quaternary ammonium nitrogen atoms (C_18_H_37_-N^+^(CH_3_)_2_-C_6_H_12_-N^+^(CH_3_)_2_-C_6_H_12_-N^+^(CH_3_)_2_-C_18_H_37_, C_18-6-6-18_) [39]. When C_18-6-6-18_ is used as a template, an MCM-41 like hexagonally ordered mesoporous material was prepared. Transmission electron microscopy (TEM) results indicated that the mesopore wall of the sample is fully crystalline with an MFI framework structure. Two-dimensional hetero-nuclear correlation nuclear magnetic resonance spectroscopy (2D HETCOR NMR) further verified the role of N-contained amphiphilic quaternary ammonium surfactants for the preparation of these hierarchical zeolites. On one hand, strong interactions between quaternary ammonium nitrogen and framework silicon infer that hydrophilic head of molecule promotes the crystallization of a zeolite framework. On the other hand, the lack of correlated signal intensity between ^29^Si from MFI framework and ^1^H from hydrophobic C_18_H_37_ tail indicates that the alkyl tail is not molecularly proximate in the framework. By modifying the molecular structure of the amphiphilic surfactants, the properties of the zeolite framework or ordered mesoporous structures were modified [43]. For example, by inserting phenyl into hydrophilic head, a BEA type framework was formed. For MCM-41 like mesostructure with fully crystalline pore wall, the thickness of mesopore wall and mesopore diameter can be adjusted by changing the number of quaternary ammonium nitrogen atoms and the length of alkyl group length, respectively. Compared with conventional MCM-41, the sample templated by C_18-6-6-18_ exhibited superior performances in alkylation and acylation of bulky reactants.

In the discussion above, the recruited amphiphilic surfactants are multi-head quaternary ammonium molecules due to unfavourable thermodynamics. Che *et al.* introduced bi-phenyl or naphthyl groups into the hydrophobic moieties of single-head quaternary ammonium surfactant to guide the synthesis of house-of-cards-like arrangements with perpendicularly interconnected single-crystalline mesostructured MFI nanosheets [44]. The hydrophilic quaternary ammonium heads are located at straight channels and promote the formation of a MFI framework, while the hydrophobic tails separate the neighbouring zeolite sheets. Further characterizations confirmed that the structures are stabilized via the π-π stacking interactions between the adjacent molecules (Fig. 3e).

### Auxiliary mesopores

**Figure 4.**
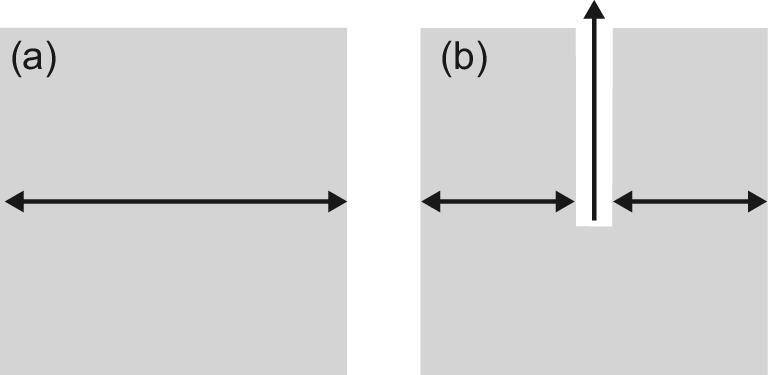
Decreased diffusion length (shown as double-headed arrows in the figure) in the micropores within zeolite crystals via auxiliary mesopores: (a) without mesopores and (b) with auxiliary mesopore inside the zeolite crystal. Re-drawn and adapted with permission from [45].

Differently to functional mesopores, introducing auxiliary mesopores cannot prevent intracrystalline microporous diffusion, or in other words, the acid sites are still located within the traditional crystalline microporous frameworks. As shown in Fig. 4, auxiliary mesopores provide shortcuts to diffuse from the outer surface of zeolite crystals to the surface of mesopore walls [45]. Therefore, for such hierarchical zeolites, the improvement of the catalyst effectiveness factor mainly comes from shortening the intracrystalline microporous *L*.

The synthesis methods applied toward preparation of materials with auxiliary mesopores are divided into two categories based on the stages of forming micro- and mesopores: (i) post-treatment methods, in which the micro- and mesoporous structures are formed separately; and (ii) *in situ* methods, in which the two types of porous structures are formed simultaneously.

### Post-treatment methods: distinct formation of micro- and mesoporous structures

**Figure 5.**
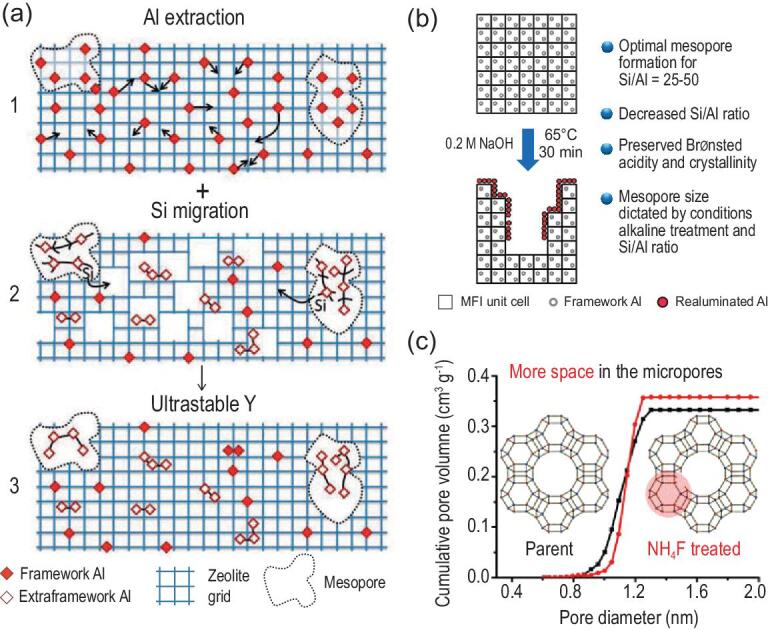
Representative auxiliary mesopores formed via post-treatment strategies. (a) Mechanism of formation of secondary mesopores via dealumination. Adapted with permission from [46]. (b) Properties of desilication method. Adapted with permission from [48]. (c) Opening the cages of FAU zeolite via fluoride-mediated post-treatment. Adapted with permission from [55].

The post-treatment strategy involves selective removal of atoms from the zeolite framework via acid or base treatments. Based on the type of the removed atoms, the strategy can be described as dealumination (Fig. 5a), desilication (Fig. 5b) and fluoride-mediated post-treatment (Fig. 5c). The mechanism of dealumination is shown in Fig. 5a [46]. The dealumination process causes breaking of Si-O-Al bonds and expulsion of aluminium species and formation of vacancies that may cause partial amorphization of the zeolite structure. The local amorphous structure is a medium with high migration properties, causing some of the defect sites of the aluminium atoms to be re-filled while others continue to grow to form mesoporous holes. In some places where the dealumination is more intense, or where there are many defects, a part of the spherical mesoporous holes can be further aggregated to form larger mesoporous channels. However, the mesopores generated via dealumination methods are mainly closed mesopores surrounded by a microporous zeolite framework, which cannot efficiently interconnect with the external surface of zeolites [47].

Desilication is the method where silicon atoms from a zeolite framework are selectively removed and thereby auxiliary mesoporous structures are introduced [48] (Fig. 5b). Desilication agents are usually alkaline agents capable of preferentially breaking Si-O-Si bonds [49]. Compared to dealumination, the desilication approach generates a more easily interconnected mesopore network that is directly linked to the outer surface of zeolites. Groen *et al.* reported two orders of magnitude of improvement in the average characteristic diffusion time of a controlled desilicated ZSM-5 sample [50]. Early studies revealed that the optimal Si/Al ratio of the parent zeolite is 25–50 due to the re-insertion (re-alumination) of extra-framework Al species accompanied with desilication [51]. Further, Pérez-Ramírez *et al.* [48] extended Si/Al ratio range to all Si/Al ranges via either co-incorporating metal salts or organic alkylammonium salts (Si/Al > 50), or adopting acid washing prior to desilication (Si/Al < 25).

Fluoride-mediated post-treatment is based on a simultaneous removal of Si- and Al-containing species. Valtchev *et al.* first proposed a generalized strategy to prepare hierarchical zeolite with auxiliary mesopores, using HF-NH_4_F buffer solution as post-treatment agent [52,53]. NH_4_F inhibits the dissociation of HF to form H^+^ and F^−^ ions and promotes the combination of HF and F^−^ ions concurrently. The overall result is the formation of HF_2_^−^ ions, which can hydrolyse indiscriminately both Si-O-Si and Si-O-Al bonds [54], therefore keeping a constant Si/Ai ratio of the samples during the whole post-treatment process. Qin *et al.* applied the fluoride-mediated post-treatment method in FAU zeolite and found that the process can selectively open the sodalite cages of FAU zeolite without causing the collapse of the supercage [55] (Fig. 5c), indicating that the HF-NH_4_F buffer solution enables zeolite framework post-treatment with atomic-level accuracy.

### *In situ* strategy: simultaneous formation of two types of porous structures

**Figure 6.**
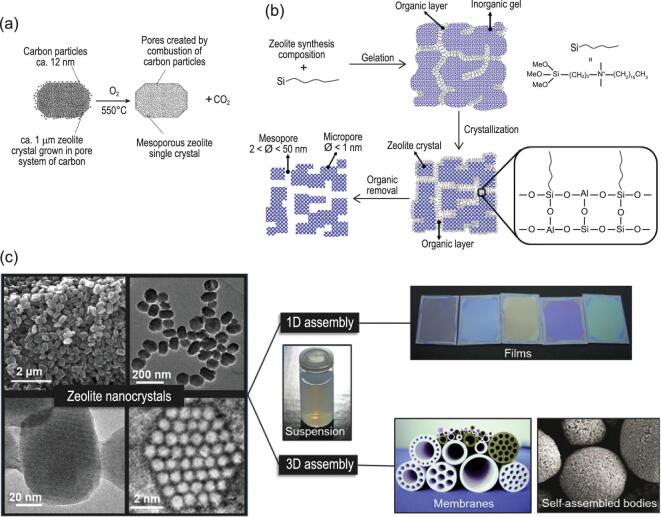
Representative auxiliary mesopores formed via *in situ* strategies. (a) Mesopores formed in ZSM-5 zeolite via carbon template. Adapted with permission from [57]. (b) Formation of mesopores via organosilane surfactant template. Adapted with permission from [43]. (c) Nanosized zeolite crystals with a diverse morphology and size, synthesized from colloidal suspensions, self-supported shapes, porous membranes and optical quality films. Adapted with permission from [63].

Using the *in situ* strategy, the crystalline zeolite framework and the auxiliary mesoporous structures are simultaneously generated. Based on the templates used for auxiliary mesopore formation, the *in situ* strategy can be divided into hard-template process (Fig. 6a), soft-template process (Fig. 6b) and template-free process, i.e. direct synthesis of nanosized zeolites (Fig. 6c).

Hard-templates only act as mesopore filler during the crystallization of zeolites. Typical hard-templates are carbon materials [56], which have versatility to be applied for a variety of frameworks (Fig. 6a) [57–60]. The soft-templates like polymers or supramolecular micelles usually interact with Si- and Al-species via covalent, hydrogen bonds or Coulomb forces. In comparison to hard-templates, soft-templates are more flexible in controlling the mesopores formation. A typical example of soft-template is an amphiphilic organosilane ([3-(trimethoxysilyl)propyl] hexadecyl dimethylammonium chloride ([(CH_3_O)_3_SiC_3_H_6_N(CH_3_)_2_C1_6_H_33_]Cl, TPHAC)) designed by Ryoo *et al.* (Fig. 6b) [43,61]. TPHAC consists of a surfactant-like long-chain alkylammonium moiety and a hydrolysable methoxysilyl moiety. Because the two parts are connected by Si-C bonds, the chemical stability of the template is preserved under zeolite synthesis conditions, and the separation of zeolite and mesopore phases is avoided. The mesoporous zeolites showed low coke deposition rate and more extended lifespan in the methanol-to-hydrocarbon reaction [62].

Adopting a template-free process is based on the direct preparation of zeolite crystals with nanoscale size and shape (Fig. 6c). The particle sizes of zeolite crystals are reduced to nano-dimensions by controlling the nucleation and growth conditions of the zeolite [63,64]. With a decrease of the zeolite crystal size, the ratio of external to total specific surface area is significantly increased, which allows more active sites to be accessible. In addition to the increased accessibility of the acid sites, the diffusion length of reactive species is shortened. The nanosized zeolites exhibit excellent performances in reactions such as liquid phase synthesis of ethylbenzene [65,66] and hydroisomerization of linear alkanes [67]. Mintova *et al.* have been committed to the fabrication of zeolite materials with different frameworks structures with and without an organic template [63,64,68–72]. Nanosized FAU zeolite with uniform crystal size (10–15 nm), and variable Si/Al ratios (1.1–2.6), high micropore pore volume and specific surface area over 800 m^2^/g showed great performance in tri*-*iso*-*propylbenzene cracking reaction [72].

### Integrated mesopores

**Figure 7.**
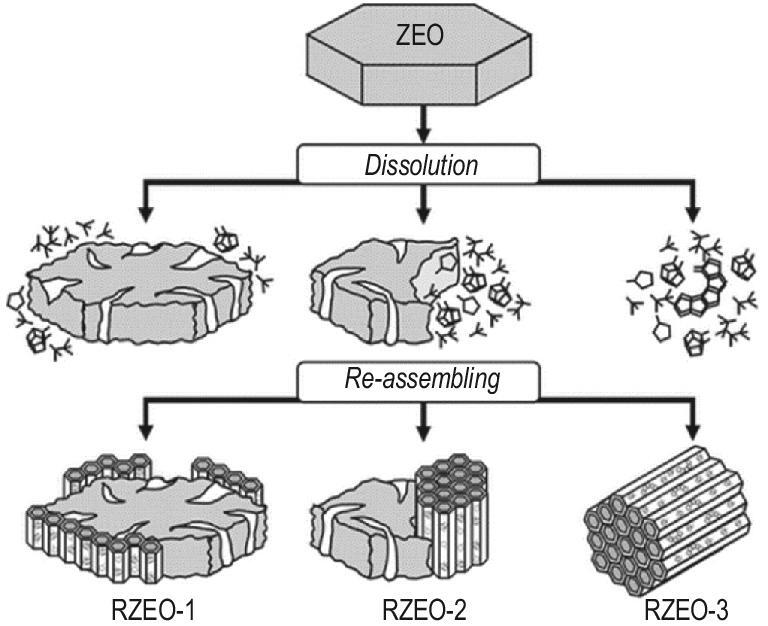
Schematic representation of synthesis procedure leading to different types of integrated mesopores. Adapted with permission from [78].

Hierarchical zeolite with integrated mesopores is a porous system where both functional and auxiliary mesopores are present. The basic idea of introducing an integrated mesopore is to sequentially couple the alkali dissolution with self-assembly of ordered mesoporous structures. The initial synthesis step of preparing integrated mesopore-containing hierarchical materials is the alkali dissolution of conventional microporous zeolite, which can generate the auxiliary mesopores in the zeolite framework. At the same time, desilication of the zeolite framework is accompanied by the loss of silica-aluminium species. These aluminosilicate species are usually amorphous nanoparticles that still maintain the primary and secondary building units of zeolite [73]. Using a template-like CTAB, they can further form ordered mesoporous structures and overgrow on the surface of the retained mesoporous zeolite crystals. Due to the coupling of functional mesopores and auxiliary mesopores, the integrated mesopore-containing hierarchical zeolite can simultaneously shorten the diffusion path of reactive species and increase the effective diffusion coefficient [74]. Alkali dissolution level is the most important factor tuning the textural and acid properties of the hierarchical zeolites [75–77]. Ivanova *et al.* classified the hierarchical zeolites with integrated mesopores into three distinctive types [78] (Fig. 7). Peng *et al.* further showed that the decrease of the zeolitic microporous structure and the increase of the ordered mesoporous structure do not change linearly with the increase of alkalinity of treating solutions [79]. The transition from one to another structure only occurs if critical alkali concentration is achieved. Alkalinity less than this critical concentration does not cause collapse of the framework, but only leads to partial damage of the zeolite framework. Recently, by making full use of the knowledge on the process control of the sequential alkali dissolution and mesopore reassembly, a pilot scale-up synthesis of hierarchical zeolites (50 L) with high stability, reproducibility and similar fluid catalytic cracking (FCC) performance was realized by Yan *et al.* [80].

### Influence of hierarchy at zeolitic level on catalyst effectiveness

**Figure 8.**
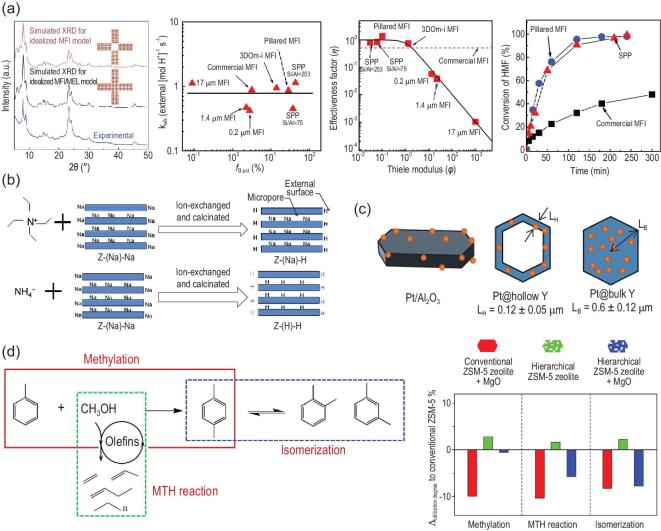
Examples demonstrating the influence of hierarchy at zeolitic level on catalyst effectiveness. (a) Experimental and simulated X-ray diffraction patterns for zeolite framework structures and comparison of benzyl alcohol alkylation performance of self-pillared pentasil nanosheets with pillared, 3DOm-i, commercial and conventional (0.2, 1.4 and 17 μm) MFI zeolites. Adapted with permission from [40]. (b) Method to differentiate the origin of enhanced catalyst efficiency by ion-exchange with bulky (i.e. tetra propyl ammonium TPA^+^) and/or small cations (i.e. ammonium NH_4_^+^). Adapted with permission from [81]. (c) Schematic representation of various materials and their corresponding characteristic length. Adapted with permission from [82]. (d) Mass transfer advantage of hierarchical zeolites promotes methanol converting into para-methyl group in toluene methylation. Adapted with permission from [83].

As one of the core aims of constructing a hierarchical structure in zeolitic components, enhancement of the catalyst effectiveness always goes hand in hand with the development of synthesis strategies. For example, Tsapatsis *et al.* reported self-pillared pentasil (SPP) zeolites via repetitive branching [40,41]. Experimental and simulated X-ray diffraction patterns showed that the 90^o^ rotationally arranged single-unit-cell MFI nanosheets are the main building blocks to form a house-of-cards arrangement of the sample, while needles of MEL serve as conjunctions to connect the rotated nanosheets (Fig. 8a). Compared with conventional microporous ZSM-5, the hierarchical material had more Brønsted acid sites exposed on the external surface of SPP zeolite. Due to the increased accessibility of acid sites, both the pseudo-first-order rate constant and the effectiveness factor of SPP zeolite are much higher than that of traditional ZSM-5 (Fig. 8a). However, the rate constant normalized per external acid site for either SPP zeolite or conventional ZSM-5 sample is nearly the same, and the catalytic properties (Brønsted acid sites) in these two samples are comparable. It means that the intrinsic reaction rate is almost constant.

Since the improvement in catalyst effectiveness can be attributed to raised effective diffusivity and/or reduced diffusion length, it is necessary to unveil the origin of enhancement. Xie *et al.* proposed a facile method to differentiate the above-mentioned two distinctive origins [81]. The pre-synthesized Na^+^-type hierarchical zeolites were ion-exchanged with NH_4_^+^ and/or tetra propyl ammonium cation (TPA^+^). TPA^+^ can only exchange with the Na^+^ cation located on the surface of external (mesoporous) active sites due to steric effect on the inherited MFI microporous framework, while the size of NH_4_^+^ is small enough to guarantee the ion-exchange with all the Na^+^ cations wherever they are located (Fig. 8b). The kinetic analysis showed that the activation energy of both TPA^+^ exchanged hierarchical and conventional MFI zeolite are nearly the same, but activation energy of the samples after ion-exchange with NH_4_ is different. This phenomenon elucidates that the enhancement of catalyst effectiveness originates from the shortened diffusion length, rather than raised effective diffusivity. Farrusseng *et al.* reported the hierarchical Pt/Na FAU zeolite with mesoporous hollow morphology, which does not alter the external surface of the crystals [82]. In this sense, this material can be regarded as a model catalyst where the increased catalyst effectiveness solely comes from reduced diffusion length (Fig. 8c). The catalytic performance and catalyst effectiveness of the cyclohexene hydrogenation reaction were compared with its microporous counterpart. The result demonstrates that the Thiele modulus is descended from 1.41 for microporous Pt/Na FAU zeolite to 0.28 for the hierarchical sample, indicating an improved catalyst effectiveness from 63% to 97%.

Except for the direct impact on catalyst effectiveness, the selectivity of targeted reaction can also be controlled via hierarchization of structures due to the different diffusion behaviour of reagents and products. For example, in toluene-methanol para-methylation reaction, another two main side reactions, i.e. methanol-to-hydrocarbons (MTH) and isomerization, mightily undermine the selectivity of the targeted para-methylation reaction (Fig. 8d). Although the diffusion limitations in the reactions within the network are all influenced by hierarchical structure, the methylation reaction is more prone to diffusion property change (Fig. 8d). Besides, the modification of the micropore mouth and external surface coverage brought by MgO addition are more influential to methylation and MTH rather than isomerization. By making full use of the above effects, MgO-modified hierarchical FAU zeolite catalyst was designed by Zhou *et al.*, avoiding the occurrence of MTH and isomerization reactions thus ensuring high selectivity of the targeted para-methylation reaction (from 34.9% to 86.2%) [83].

### Pore interconnectivity at hierarchical zeolitic constituent level

Although the enhanced catalyst effectiveness of hierarchical zeolite comes from the increase of *D*_eff_ and/or the reduction of *L*, introducing functional and/or auxiliary mesopores into zeolitic constituent cannot always lead to the reduced diffusion resistance and subsequent enhanced catalytic conversion and selectivity. The work reported by Pérez-Ramírez *et al.* is a typical example [84]. N_2_ physisorption and *in situ* Fourier transform infrared spectroscopy (FTIR) using pyridine indicate that two hierarchical zeolites prepared with a shorter stronger and a longer milder treatment recipe only have negligible differences on their textural and acidic properties. Hence, these two samples showed distinctive lifetimes during the methanol-to-hydrocarbon reactions. An advanced positron annihilation lifetime spectroscopy (PALS, *vide infra*) confirmed that compared with the shorter stronger treated sample, the longer and milder treatment can ensure better micro- and mesopore interconnectivity. Good pore interconnectivity assures the fast desorption and diffusion of products generated on the microporous active sites and avoids the deposition and deactivation [62,85]. For the reactions like FCC, which always occur in a cascade manner, an ideally interconnected hierarchical porous material, that supports the concept of ‘hierarchical catalysis’ [86,87], is also highly appreciated. When a bulky feedstock diffuses into such hierarchical porous structure, it first contacts with the large pores and ‘pre-cracks’ by mild acid sites. Then the intermediates further diffuse into small pore structures and are cracked by strong acid sites. However, such hierarchical zeolitic pore engineering needs elaborate design of pore sizes and interconnections. Inspired by natural biological systems, Su *et al.* recently prepared porous materials with connected pores of multi-scale diameters from macro to micro levels based on ‘generalized Murray's Law’, which describes that the porous network in nature always tends to form the structure with minimum transport resistance within finite volume [88]. These materials showed improved performances in many realms where hierarchy is required.

The analysis of hierarchical porous structures acquires quantitative understanding of improved accessibility and correlation with their catalytic performances. Hitherto, many spectroscopic and microscopic techniques have been applied to unveil the connectivity between microporous and mesoporous structures.

Gas adsorption-desorption characterization is a standard technique for investigation of porous materials. Architecture and interconnectivity of hierarchical zeolites was quantitatively measured via hysteresis scanning measurements coupled with advanced simulation models for calculating pore size distribution [89–92]. A sorption isotherm was acquired routinely as ‘boundary adsorption/desorption isotherm’. Then the isothermal adsorption is re-conducted at a relative pressure where pores are partially filled (at the hysteresis loop). If the shape of the desorption scanning curve coincides with that of the boundary desorption branch, this means that the measured mesopores are occluded. On the contrary, the deviation of the desorption scanning curve from the boundary desorption branch infers the onset of pore block/percolation, which is a sign that the tested porous structures are interconnected. Via differential hysteresis scanning, Kenvin *et al.* quantitatively calculated the relative amount and size of pyramidal (d_meso_ > 2 nm, d_win _> d_meso_), constricted (d_win_ > 2 nm, d_win_ < d_meso_) and occluded (d_win_ < 1 nm, d_win_ << d_meso_) mesopores in hierarchical FAU zeolites [92]. The results showed that 90% of the generated mesopores in FAU zeolites after steaming are occluded or constricted. Mild acid treatment promoted the formation of pyramidal structures because aluminium-rich debris are washed away by acid, but more severe acid treating led to more extensive dealumination resulting in an enhanced portion of occluded mesopores. In terms of the subsequent desilication, the fraction of pyramidal mesopores increases with the increased alkalinity.

Accessibility measurements were performed via *in situ* FTIR using a series of probe molecules with varied kinetic diameters [93]. The adsorption behaviour of several (alkyl)pyridine molecules on conventional and hierarchical ZSM-5 via *in situ* FTIR spectroscopy was described in detail [94]. The pyridine (Py) with a kinetic diameter of c.a. 0.57 nm, which is close to the size of micropores of ZSM-5 zeolite was used to study the acid sites. In contrast, bulky probe molecules, 2,6-lutidine (Lu) and 2,4,6-collidine (Coll), with larger kinetic diameters of 0.67 and 0.74 nm, respectively, were used as probe molecules to study the acid sites accessible via the mesopores. The results revealed the increased adsorption of Lu and Coll in desilicated hierarchical zeolites. Based on these results, an accessibility index (ACI), a descriptor standardized for illustrating acid site accessibility in ZSM-5 with mesopores, was derived [48,94]. Since both zeolitic Brønsted and Lewis acid sites are detectable by pyridine, the ACI of pyridine can be calculated using the following equation:

(7) \begin{equation*} \mathit {ACI}{_{{\rm{Py}}}} = \frac{{({C_{\rm{B}}} + {C_{\rm{L}}})}}{{{n_{{\rm{Al}}}}}}, \end{equation*}

where *ACI*_Py_ is the accessibility derived from pyridine; *C*_B_ and *C*_L_ are the amount of Brønsted and Lewis acid sites from pyridine, respectively; and *n*_Al_ is the total amount of acid sites in the zeolite based on the measured aluminium content.

In the case of 2,6-lutidine and 2,4,6-collidine that only detect Brønsted acid sites, the equation is modified as follows:

(8) \begin{equation*} \mathit {ACI}{_{{\rm{Lu\ or\ Coll}}}} = \frac{{{C_{\rm{B}}}}}{{{n_{{\rm{Al}}}}}}. \end{equation*}

In addition to *in situ* FTIR, pulsed field gradient NMR (PFG-NMR) was used to calculate the self-diffusivity in hierarchical materials [95]. In the PFG-NMR experiment, sequences of strong field gradient pulse were required. Since the diffusivity is directly obtained from the square root of displacement of the probe molecule, this method is particularly suitable for determination of the diffusion coefficient in fast diffusion systems. Kärger *et al.* investigated the intracrystalline diffusion in ultra stable zeolite Y (USY) [96]. Their results showed that the disconnected secondary mesopore network cannot efficiently enhance the diffusion properties of USY zeolite. In contrast, Galarneau *et al.* showed a great increase in diffusivity in hierarchical FAU by post-treatment method compared with the parent zeolite [97].

Electron tomography (ET) is used to reconstruct the micro-/mesoporosity of hierarchical zeolites and visually showed the connectivity and accessibility of pores [98,99]. De Jong *et al.* used three-dimensional transmission electron microscopy to study the accessibility of mesopores in USY samples. The results showed that the mesopores generated via steaming were mainly closed cavity surrounded by a microporous zeolite framework, which cannot efficiently connect with the external surface of USY zeolite [47,100].

**Figure 9.**
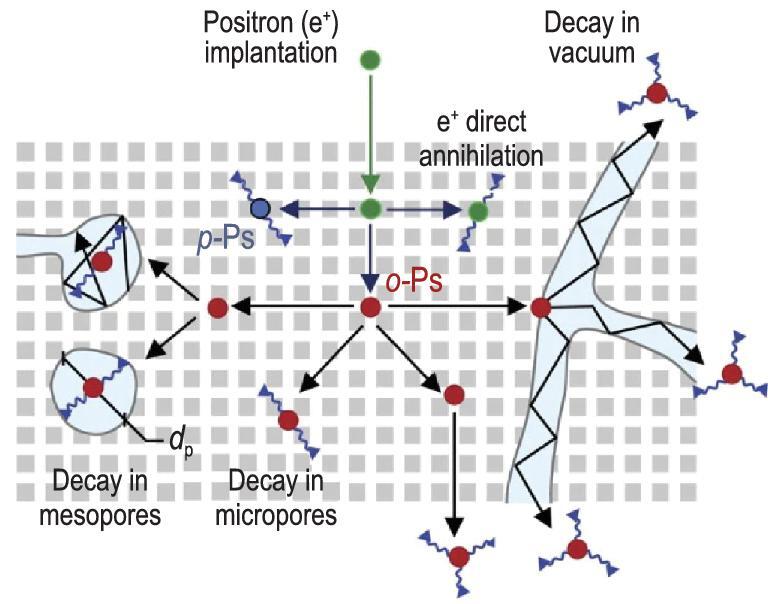
Schematic presentation of positron annihilation lifetime spectroscopy (PALS). Adapted with permission from [105].

Positron annihilation lifetime spectroscopy (PALS) recently developed, was used for quantitative evaluation of mesopore connectivity [101,102]. The positron is the anti-particle of the electron, and when a targeted material is bombarded by positron beam, most of the positrons are directly annihilated and form gamma rays. A small portion of positrons can have an extended lifetime by binding with electrons and forming positronium. There are two types of positronium: para-positronium (p-Ps) with a lifetime of ∼0.125 ns and ortho-positronium (o-Ps) with a lifetime up to 142 ns. o-Ps can diffuse into the porous structure of zeolite and its lifetime is dependent on atoms and electrons passing by. The decay time of o-Ps is proportional to the pore diameter of the tested sample; by measuring the fraction of o-Ps with different decay time, the amount of micro- and mesopores were estimated (Fig. 9) [103,104]. Milina *et al.* applied PALS to investigate conventional and hierarchical ZSM-5 with open or closed mesopores [105]. They found that the trend observed for the relative fraction of o-Ps in vacuum (i.e. free o-Ps detected outside zeolite crystals) was consistent with the lifetime trend of the catalysts in methanol-to-hydrocarbon (MTH) test reaction. Considering that only the o-Ps diffuses from intrinsic micropores via auxiliary mesopores then from the outer surface of hierarchical zeolite to the vacuum, the o-Ps was used to measure the interconnectivity between micro- and mesopores. The fraction of the o-Ps formed, which is emitted into vacuum, is defined as a descriptor called global pore connectivity (C_pore_); the C_pore_ descriptor is consistent with the MTH lifetime of the hierarchical zeolites [84].

## HIERARCHICAL PORE CONNECTIVITY IN MULTI-COMPONENT ZEOLITE BASED CATALYSTS

The hierarchical porous structures described above are confined within single-component powder zeolite material. However, in real industrial catalytic processes, some additional requirements that are much more rigid than those at laboratory test-scales should be satisfied to endow the industrial catalysts with high mechanical strength, hydrothermal stability and resistance to poisoning and coking [106–110]. Apparently, the above-mentioned requirements cannot be solely fulfilled by single-component zeolite catalyst powder. Although the inter-component interactions within the industrial catalyst body is still not fully undetrstood, the interactions between zeolitic and non-zeolitic components can result in: (i) a change of the hierarchical pore structure; (ii) framework atoms transfer; and (iii) balancing cations transfer [18]. In this review, we only focus on the first type of interaction, while for the other two types, please refer to some other references such as [18] and [111].

### Importance of different components’ locations and interconnectivity for their catalytic performance

The multi-component zeolite based catalyst is in essence different from a mechanical mixture of individual ingredients [97,112]. Therefore, the physical and chemical properties of a multi-component zeolite based catalyst not only depend on the relative amounts of constituent porous components, but also on the location and connectivity between them [19]. Below, the focus will be on the effects of location and interconnectivity of porous components.

**Figure 10.**
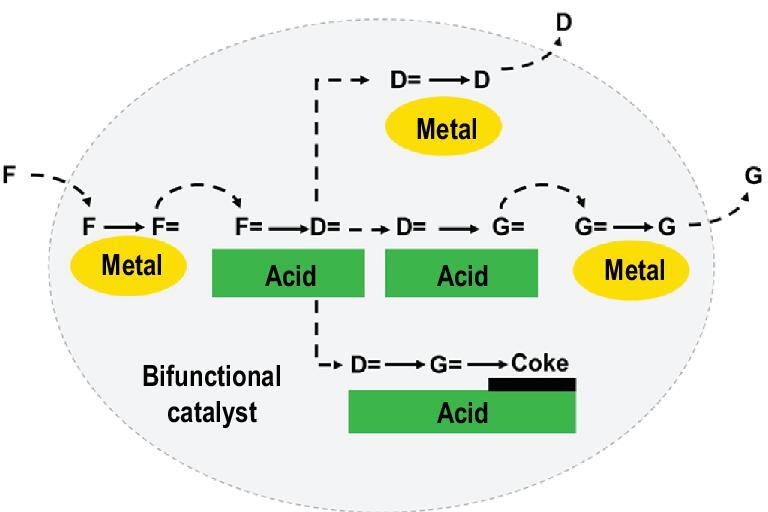
Scheme of hydrocracking reactions that use a bifunctional catalyst. Re-drawn and adapted with permission from [116].

The influence of the location of the porous components on the catalytic performance is a consequence of the distance between the different active sites located on different matters. The bi-functional hydrocracking catalyst is a typical example. In order to suppress the fast deactivation, enhance the selectivity, and operate the process under mild condition, metal active sites are often combined with Brønsted acid sites in the bi-functional catalyst. When n-alkane is adopted as feedstock (Fig. 10), the feed n-alkanes are first dehydrogenated on the metal surface to form n-alkene intermediates. Then the n-alkene intermediates diffuse from the metal sites to Brønsted acid sites where these n-alkene intermediates are either skeletally isomerized or further cracked. Then these isomerized or cracked n-alkene intermediates diffuse to metal sites and hydrogenate into isomerized or cracked products. Typically, metal nanoparticles are located on the surfaces of zeolitic and/or non-zeolitic supports [67,113], while the Brønsted acid sites are usually originated from zeolites (e.g. USY and β zeolites), and alumina acts as binder. An ideal bi-functional catalyst should show a balance between the (de)hydrogenating and acid functions. The intimacy between metal and acid sites affects the conversion and selectivity of the feedstock since it dominates the diffusion of olefinic intermediates between the two types of active sites [114]. For a long time, in terms of the intimacy of metal and acidic active sites, the criterion ‘the closer the better’ was regarded as the rule of thumb [115]. However, recent research proposed by de Jong *et al.* violated the above golden rule. In their research, Pt nanoparticles were impregnated on the surface of a support in which Y zeolite and alumina were intimately mixed at a nanoscale level [116]. By using Pt(NH_3_)_4_(NO_3_)_2_ and H_2_PtCl_6_·6H_2_O as precursors, Pt nanoparticles were deposited exclusively either on the zeolite (denoted as Pt-Y/A) or alumina (denoted as Pt-A/Y), respectively. In this way, Pt nanoparticles were purposely located either in the closest proximity to the zeolite acid sites or apart from the zeolitic acid sites at a nanoscale distance. Surprisingly, when n-decane and n-nonadecane were chosen as model hydrocracking feedstock, the Pt-A/Y sample outperforms Pt-Y/A sample in terms of high isomerization yield and limited secondary cracking products. A plausible explanation provided is the diffusion limitation of the olefinic intermediates. Due to the low diffusivity inside the micropores, olefinic intermediates generated via the encapsulated Pt are largely trapped within the zeolite framework of Pt-Y/A and undergo successive secondary cracking. In contrast, the olefinic intermediates in Pt-A/Y freely desorb from the surface of Pt nanoparticles, diffuse to the zeolitic acid sites, and only interact with the acid sites located at the external surface of zeolites. In this regard, design of the zeolitic acid sites location and metal sites to guarantee an appropriate distance between them is a key issue to be considered in future research.

**Figure 11.**
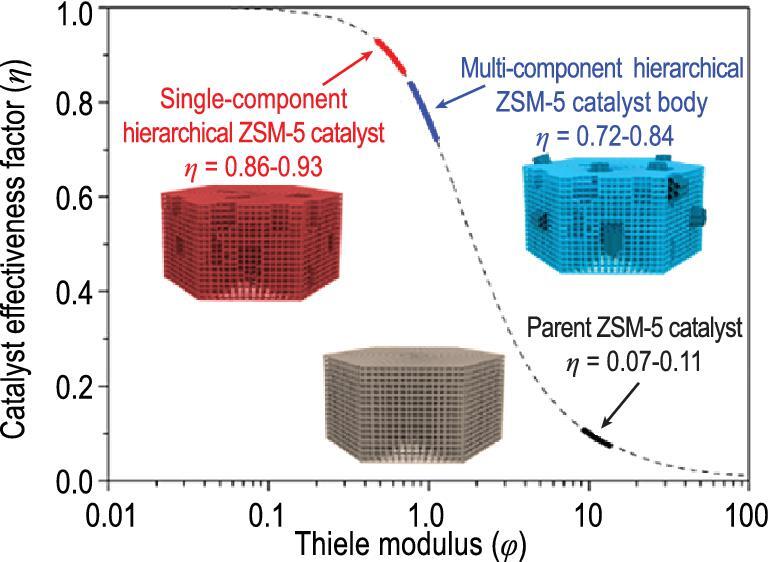
The non-ideal matching of the hierarchical pore structure between the zeolite and non-zeolite components leads to a decrease in the catalyst effectiveness factor. Adapted with permission from [120].

Another classical case to show the importance of different components’ locations and interconnectivity for their catalytic performance is FCC catalyst. Since the traditional FCC catalyst shaping process chiefly consists of pre-synthesized zeolitic and non-zeolitic components, their proportions can be finely controlled, making the whole process flexible. The contemporary suppliers of FCC catalysts can even customize materials according to the specific conditions of different refineries. However, FCC catalysts made by traditional shaping process are still mechanical mixtures of components due to non-homogeneous mixing [117]. A newly developed *in situ* crystallization shaping method, where the zeolitic component is *in situ* crystallized on the surface of the pre-treated matrix component, can ensure strong connection and interaction between the components at nanoscale [118,119]. However, the intimate connection of zeolite and non-zeolite components at nanoscale does not guarantee the ideally coordinated connection of the hierarchical porous structures from different catalyst components. Peng *et al.* recently used isooctane as a probe molecule combined with gravimetric analysis and FTIR spectroscopy (AGIR) to measure the enhancement of catalyst effectiveness brought by introducing integrated mesopores to form a model multicomponent zeolite based catalyst via sequential alkali dissolution of a commercial ZSM-5 followed by Al-MCM-41 mesostructure reassembly. The results show that due to the non-ideal arrangement of mesoporous ZSM-5 component (representative of zeolite components) and Al-MCM-41 component (representative of non-zeolite components), the promotion effect of the catalytic efficiency factor of zeolite components is greatly reduced (Fig. 11) [120].

### Visualizing the locations and pore interconnectivity of components within an individual zeolite based catalyst body

As discussed above, the relative positions of the different types of active sites on components may have positive or negative effects on the performance of the catalysts [116,121–123]. There has always been strong motivation to explore and visualize the relative positions of active site distributions. Nitrogen (or argon for more precise measurements) adsorption-desorption isotherms and mercury porosimetry are the most applied characterization techniques for pore size distribution and textural properties assessment of heterogeneous catalysts. However, as macroscopic tools, the data derived from these two methods only reflect the average of massive catalyst particles [117]. Considering that the catalyst behaviour at single-particle level shows many similarities with the life activities within a single cell [124], some bio-microscopic methods were adopted to locate different components within a single catalyst body. Besides, thanks to the rapid advancements in the field of microscopic techniques where optical light, X-rays and electrons are used as probes based on synchrotron radiation techniques, the (operando) exploration and analysis of catalyst particles at individual particle scale has become available [125,126]. In this section, two typical cases will be presented as examples for showing the state-of-the-art on visualizing the locations and pore connectivity of components within an individual zeolite based catalyst body.

**Figure 12.**
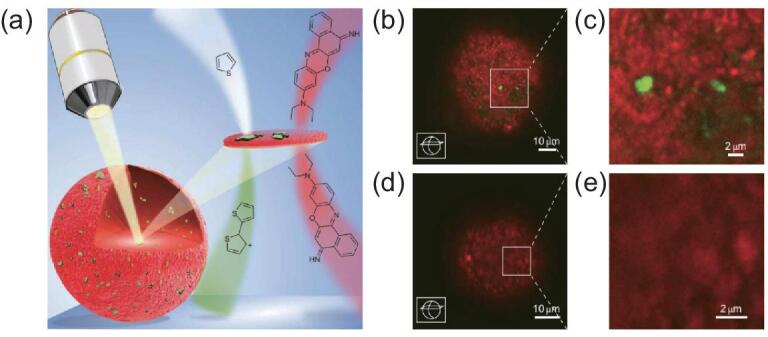
Schematic of the research approach. (a) Confocal fluorescence microscopy is used to visualize distinct components of fluid catalytic cracking (FCC) catalyst particles after staining with thiophene (green) and Nile Blue A (red); confocal fluorescence microscopy image of the stained FCC industrial catalyst bodies with (b and c) and without (d and e) zeolite Y. Adapted with permission from [127].

Buurmans *et al.* developed a selective staining methodology for determining the position of zeolitic and non-zeolitic components of FCC catalyst (Fig. 12) [127]. In this research, two probe molecules, thiophene and Nile Blue A, were used. Thiophene can be oligomerized by the Brønsted acid sites on zeolitic component at a suitable temperature, and the formed oligomer can be excited by a 488 nm laser to generate green fluorescence. On the other hand, Nile Blue A, which is inert to Brønsted acid site catalysed reaction, can emit red fluorescence under excitement of a 638 nm laser. Because the kinetic diameter of Nile Blue A is larger than the micropore of zeolite, they can be only adsorbed on the external surface of zeolite crystals. The locations of zeolitic and non-zeolitic components in FCC catalyst can be identified by simultaneous stain of thiophene and Nile Blue A and image by confocal fluorescence microscopy. Based on the selective staining method, three additional samples treated by three simulated deactivation methods (steam (ST), two-step cycle deactivation (CD), and Mitchell impregnation-steam deactivation (MI)) were characterized. The fluorescence intensity related to thiophene derivative decreased in the following order: fresh catalyst > ST > CD > MI.

**Figure 13.**
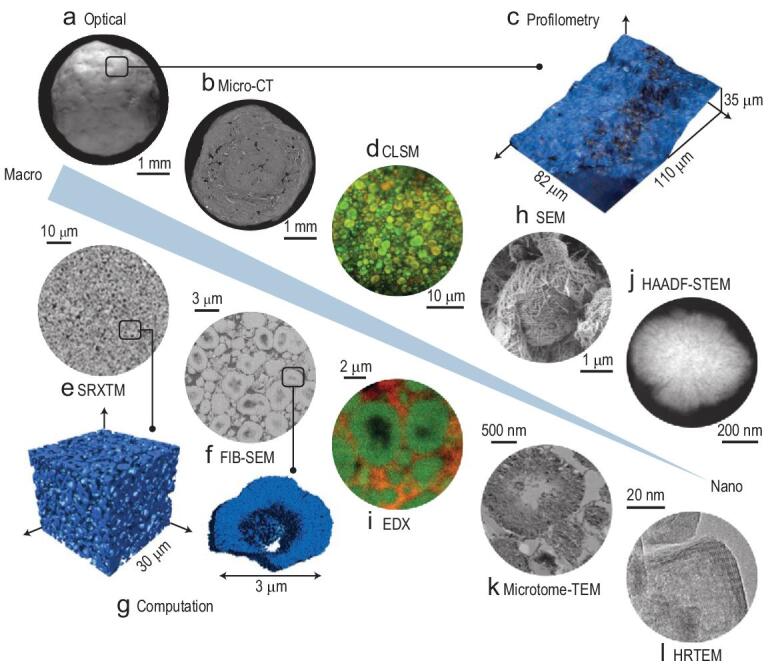
Integrated approaches for visualization of an industrial zeolite catalyst from macro- to nano-length scales. (a) Optical microscopy: macroscopic structure of a hierarchical zeolite catalyst granule; (b) X-ray micro-tomography (micro-CT): the internal structure; (c) profilometry and (d) confocal laser scanning microscopy (CLSM): external surface; (e) synchrotron radiation X-ray tomographic microscopy (SRXTM) and (f) focused ion beam scanning electron microscopy (FIB-SEM): homogeneous internal distribution of zeolite and binder phases; (g) visualized and calculated macro- and mesopore structures based on SRXTM and FIB-SEM; (h) SEM: arrangement of binder particles at the external surface of zeolite particles; (i) energy dispersive X-ray spectroscopy (EDX): elemental maps of silicon (green) and aluminium (red); (j) high-angle annular dark-field scanning transmission electron microscopy (HAADF-STEM): uniform distribution of intracrystalline mesopores within individual zeolite aggregates; (k) transmission electron microscopy (TEM) and (l) high resolution transmission electron microscopy (HRTEM): nanostructural insights of microtome cross-sections. Adapted with permission from [19].

Each spectroscopic or microscopic method brings important information and has certain limitations. As a complex system involving multi-scale physical and chemical problems, it is necessary to understand simultaneously the pore structure connectivity within a catalyst at different scales. Pérez-Ramírez *et al.* combined a series of advanced optical, X-ray and electron-based microscopic and tomographic techniques to visualize the amount, location and connectivity of mesoporous ZSM-5 catalysts from millimetre-size granules to nanosized zeolite crystals (Fig. 13) [19]. At macroscale, digital optical microscopy was used to detect the morphology and surface roughness of the shaped catalyst body. Profilometry and Confocal Laser Scanning Microscopy (CLSM), together with appropriate staining, was used to study the external surface of the catalyst. Micro-Tomography (micro-CT) and Synchrotron Radiation X-ray Tomographic Microscopy (SRXTM) were applied to investigate the location and distribution of zeolitic and non-zeolitic components in the interior of the catalyst. More precise discrimination between zeolitic and non-zeolite components in the catalyst required the use of both the Focused Ion Beam Scanning Electron Microscopy (FIB-SEM) and the Energy Dispersive X-ray spectroscopy (EDX). Then detailed information on the connection and interaction at the interface of zeolitic and non-zeolite components was unveiled via high-resolution scanning electron microscopy (SEM). Finally, TEM was used to image the nanostructural information of zeolitic component.

## CONCLUSION

The preparation, characterization and effectiveness of hierarchical zeolites are revealed. Introduction of mesoporosity in zeolites accelerates diffusion of bulky molecules to acidic active sites originally confined within the microporous frameworks. Therefore, in terms of the synthesis and application of hierarchical zeolites, it is not only necessary to balance between improvement of mass transport and retention of acidic active sites in zeolites to preserve the advantages of both micro- and mesoporous structures, but also to consider the connectivity between the porous structures. As a consequence, three types of hierarchical zeolite structures with ‘functional mesopores’, ‘auxiliary mesopores’ and ‘integrated mesopores’ are described. As descriptors, *ϕ* and *η* for the performance of hierarchical zeolites are used.

The distinctive differences of diffusion mechanism within ‘functional’ and ‘auxiliary’ mesopores and their connectivity within porous structures are revealed. Since the active sites are located on the surface of the mesoporous structure, the strategies that guarantee pore connectivity of ‘functional’ mesopores should ensure that the acidic active sites are exposed predominantly on the external surface of mesopores. In contrast, the ‘auxiliary’ mesopores should be the bridge between the zeolite framework structures and the external surface of the catalysts.

The ultimate purpose of preparing hierarchical zeolite materials is to promote their use at industrial scale. The amount, location and connectivity of pores in hierarchical zeolites plays an essential role in their catalytic performance, therefore the properties of each constituent porous component should be separately taken into consideration. The pore arrangement of the FCC catalyst is presented as an example of hierarchical material that ensures the diffusion of bulky molecules in heavy oil feedstock and also the cracking with weak acid sites in macropores, via medium acid sites in mesopores to the strong acid sites in the micropores. The rational design of industrial multi-component catalysts at both hierarchical zeolitic component and catalyst body levels significantly effects the catalyst efficiency. The advanced combined spectroscopic, microscopic and diffraction techniques toward characterization of hierarchical materials provide better understanding of the structure–property–catalysis interplay of industrially used hierarchical zeolites.
